# Evaluation of piperonyl butoxide in enhancing the efficacy of pyrethroid insecticides against resistant *Anopheles gambiae s.l.* in Ghana

**DOI:** 10.1186/s12936-017-1960-3

**Published:** 2017-08-17

**Authors:** Samuel K. Dadzie, Joseph Chabi, Andy Asafu-Adjaye, Otubea Owusu-Akrofi, Aba Baffoe-Wilmot, Keziah Malm, Constance Bart-Plange, Sylvester Coleman, Maxwell A. Appawu, Daniel A. Boakye

**Affiliations:** 1 Parasitology Department, Noguchi Memorial Institute for Medical Research (NMIMR), University of Ghana, P.O. Box LG 581, Accra, Ghana; 2 National Malaria Control Programme, Ghana Health Service, Accra, Ghana; 3 Africa Indoor Residual Spraying Programme, Ghana Project, Accra, Ghana

**Keywords:** Pyrethroids, Piperonyl butoxide, *Anopheles gambiae*, Insecticide resistance

## Abstract

**Background:**

Malaria vector control methods involving the use of pyrethroids remain the strategies being used against malaria vectors in Ghana. These methods include the use of long-lasting insecticidal nets and indoor residual spraying in many areas in Ghana. However, there is evidence that pyrethroid resistance is widespread in many areas in Ghana. Synergists have been shown to be useful in inhibiting the enzymes that are responsible for the development of resistance and hence enhance the insecticide susceptibility of *Anopheles gambiae* sensu lato (*s.l.*) in many areas. The present study investigated the effect of piperonyl butoxide (PBO) on the susceptibility status of *An. gambiae s.l.* across some sentinel sites in Ghana.

**Methods:**

Three to five day old *An. gambiae s.l.* reared from larvae were used in WHO susceptibility tube assays. Batches of 20–25 female adult *An. gambiae s.l.* were exposed simultaneously to the insecticide alone and to the PBO + insecticide. The knock down rate after 60 min and mortality at 24 h were recorded.

**Results:**

Deltamethrin and permethrin resistance of *An. gambiae s.l.* was observed in all the sites in 2015 and 2016. The mortality after 24 h post exposure for deltamethrin ranged from 16.3% in Weija to 82.3% in Kade, whereas that for permethrin ranged from 3.8% in Gomoa Obuasi to 91.3% in Prestea. A significant increase in susceptibility to deltamethrin and less to permethrin was observed during both 2015 and 2016 years in most of the sites when *An. gambiae s.l.* mosquitoes were pre-exposed to PBO.

**Conclusion:**

Findings from this study showed that the use of PBO significantly enhanced the susceptibility of *An. gambiae s.l.* mosquitoes in most of the sentinel sites. It is recommended that vector control strategies incorporating PBO as a synergist can be effective in killing mosquitoes in the presence of deltamethrin and permethrin resistance.

## Background

The development of resistance by mosquitoes to the major pyrethroid insecticides being used for malaria control still remains one of the major obstacles of the effective control of the disease and threatens to hinder efforts at malaria elimination globally and especially in Africa. The rapid spread of the insecticide resistance of *Anopheles gambiae* sensu lato (*s.l*.) throughout sub-Saharan Africa and its impact on the failure of vector control strategies has become the main worry of all the national malaria control programmes. Most of the available control tools are facing a decrease of effectiveness following increasing insecticide resistance of *An. gambiae s.l.*, the main malaria vector [1–3].

In Ghana, the use of insecticides for public health and agriculture remain the main strategy for controlling disease vectors and pests. The distribution and use of insecticide-treated nets (ITNs) as well as indoor residual spraying (IRS) have been scaled up as the main vector control tools in the country. However, resistance to pyrethroids and other classes of insecticides have been reported in parts of Ghana [4–10]. This poses a challenge to the success of most of the insecticide-based vector control tools in controlling disease vectors including those of malaria in Ghana. Therefore, there is the need to monitor the level of insecticide resistance in the country so as to be able to formulate resistance management strategies.

Recent efforts have centered on developing control strategies that can be effective even in the presence of pyrethroid resistance. Studies have shown that the use of synergist in addition to insecticides has revealed to be a good option of controlling resistant mosquitoes [11, 12]. Commonly used synergists include piperonyl butoxide (PBO), which inhibits cytochrome P450 monooxygenase enzyme activity targeting DDT and pyrethroid insecticides [13, 14]. PBO is an organic compound used as a component of pesticide formulations and as synergist. Despite having no pesticidal activity of its own, it enhances the potency of certain pesticides such as especially for carbamates, pyrethrins, pyrethroids, and rotenone [14, 15]. PBO acts as an insecticide synergist by inhibiting the natural defenses of the insects. PBO inhibits enzymes present in insects, most important of which is the mixed function oxidase system (MFO) also known as the cytochrome P450 system [16, 17].

Therefore, synergists such as PBO clearly have an important role to play in increasing the efficacy of pyrethroids when used against pyrethroid-resistant mosquitoes. Currently, only pyrethroids are recommended by the World Health Organization (WHO) for use on mosquito nets, and alternatives are urgently required as insecticide resistance is compromising the performance of pyrethroid only treated nets [2, 3]. This study investigated the role of PBO on the insecticide resistance status of *An. gambiae s.l.* collected across 20 sentinel sites in Ghana.

## Methods

### Sentinel sites

**Fig. 1:**
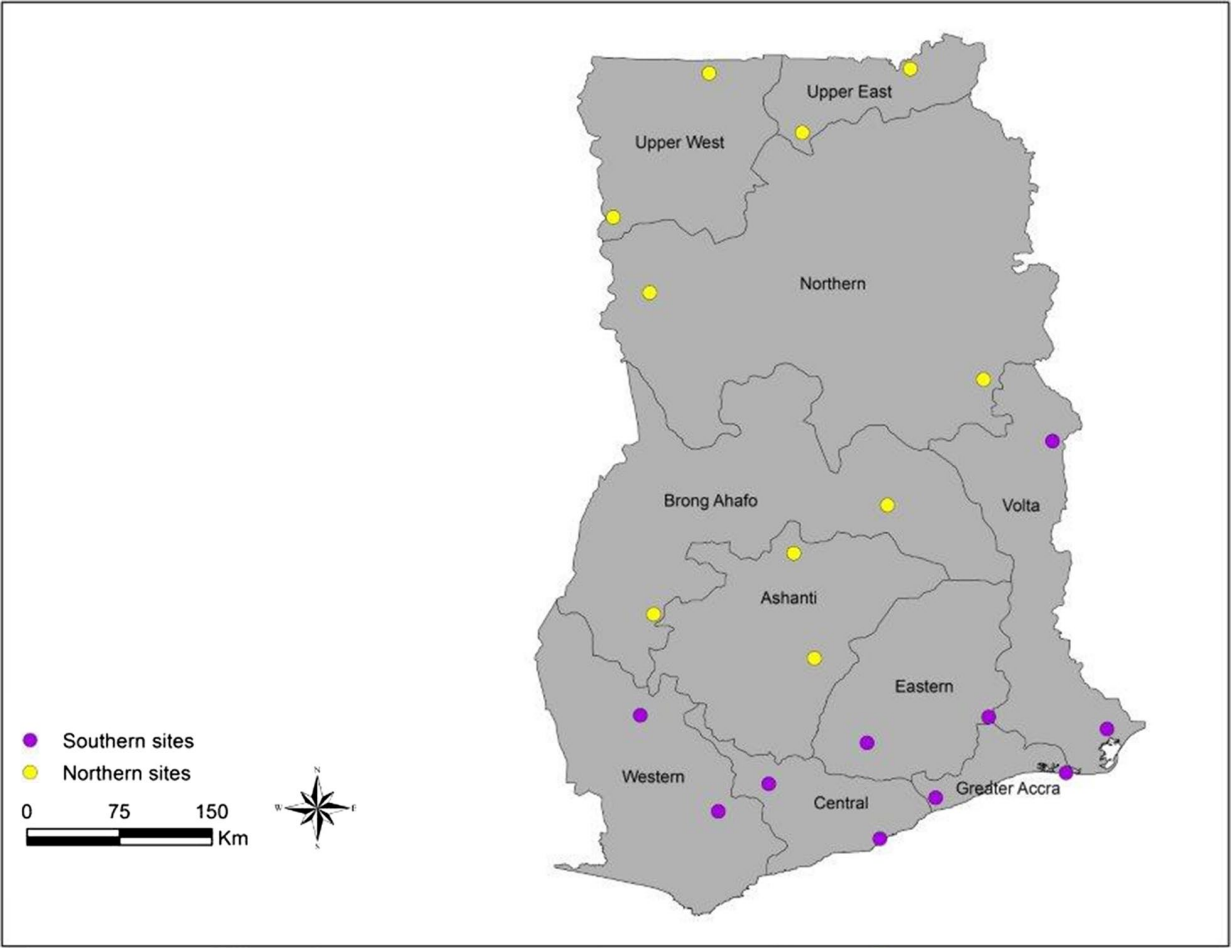
Map showing the location of mosquito collection sites across the different ecological areas in Ghana, (Southern sector sites in *purple*) (Northern sector sites in *yellow*)

Ghana is divided into ten regions and two sentinel sites were selected in each region across all the ten regions to cover all the ecological areas in the country (Fig. 1). The selection of the sites was done based on information on agricultural practices and preferentially rice and vegetable growing fields, mining and other activities that can lead to the development of insecticide resistance. The National Malaria Control Programme of Ghana (NMCP) as part of the mass campaign had also distributed pyrethroid-based long-lasting insecticidal nets (LLINs) in all the sentinel sites. For the purposes of the study, the country was divided into southern and northern sectors and also to match with the rainy season in the selected sites. Sites in the southern sector included Akuse (Lat: 6.0868, Long: 0.12139) and Kade (Lat: 6.0000, Long: −0.8332) in the Eastern Region; Nkwanta (Lat: 8.2000, Long: 0.5186) and Afife (Lat: 6.1000, Long: 0.9166) in the Volta Region; Sefwi-Wiaso (Lat: 6.2000, Long: −2.4850) and Prestea (Lat: 5.5000, Long: −1.9166) in the Western Region: Twifo-Praso (Lat: 5.6999, Long: −1.54999) and Gomoa Obuasi (Lat: 5.3000, Long: −0.7397) in the Central Region; Weija (Lat: 5.6000, Long: 0.33333) and Ada-Foah (Lat: 5.7804, Long: 0.618048) in the Greater Accra Region.

Sites in the northern sector included Sawla (Lat: 9.2833, Long: −2.4166) and Wulensi (Lat: 8.6500, Long: 0.01664) in the Northern Region; Konongo (Lat: 6.6167, Long: −1.21667) and Ejura (Lat: 7.3833, Long: −1.36667) in the Ashanti Region; Fumbisi (Lat: 10.4511, Long: −1.30595) and Zebilla (Lat: 10.9167, Long: −0.51667) in the Upper East Region; Wechau (Lat: 9.8333, Long: −2.68333) and Tumu (Lat: 10.8833, Long: −1.98335) in the Upper West Region; Kenyasi (Lat: 6.9383, Long: −2.3889) and Kwame-Danso (Lat: 7.7333, Long: −0.68333) in the Brong Ahafo Region (Fig. 1).

### WHO insecticide susceptibility bioassays

Larval collections were carried out in all the sentinel sites between the months of May to October of 2015 and 2016. The larvae were reared to adults and used for insecticide susceptibility assays according to the WHO insecticide susceptibility testing procedure [18]. Batches of 20–25 non-blood fed females of *An. gambiae s.l.* mosquitoes aged 3–5 days were introduced into each of the four tubes (replicates) and exposed to the insecticide-treated papers for an hour. Two tubes were used as controls. The temperature and relative humidity were recorded at the start and end of the testing. Knockdown rates were recorded at 5, 10, 15, 20 30, 40, 50 and 60 min and mortalities recorded 24 h after the holding period.

### Synergist assays with PBO

Whatman papers impregnated with piperonyl butoxide (PBO) synergist were used to perform the synergist assay. In total, 20–25 *An. gambiae s.l.* mosquitoes were exposed to four (4) replicates of 5% piperonyl butoxide (PBO) for 1 h to suppress oxidase enzymes. Mosquitoes were then immediately transferred and exposed to either 0.05% deltamethrin or 0.75% permethrin for an additional hour. Two control tubes were run in parallel at any time of the testing. Knockdown rates were recorded as previously for 60 min, after which the mosquitoes were transferred in holding tubes and held for 24 h after which the mortality recorded.

### Data analysis

Xlstat 2010 statistical software was used for the statistical analyses. Values were compared using the χ^2^ test and the mortalities per insecticide across site were compared using the binomial regression following the formula: f (y) = log (y/(1 − y)). The resistance status of mosquitoes was determined using the WHO criteria [19]; mortality ≤90% is resistant, mortality ≥91–97% is suspected resistant; mortality ≥98% is susceptible. All analyses were carried out at 5% level of significance and 95% confidence level.

## Results

The results of the survey indicated very high deltamethrin and permethrin resistance of *An. gambiae s.l.* in all the sites surveyed both in year 2015 and 2016. Tables 1 and 2 show the 24 h mortality rates of *An. gambiae s.l.* from the southern and northern sentinel sites exposed to both deltamethrin and permethrin alone or pre-exposed to PBO.

**Table 1:** Mortality of *Anopheles gambiae s.l.* exposed to deltamethrin and permethrin alone as well as PBO + deltamethrin and PBO + permethrin in 2015 and 2016 in the southern sector

Region	Sentinel site	Insecticide	2015				2016			
			Total tested	% Mortality	CI 95% Mortality	p value	Total tested	% Mortality	CI 95% mortality	p value
Greater Accra	Ada	Deltamethrin	80	68.8	±2.3	1.16E−13	98	4.1	±0.6	3.13E−19
		PBO + deltamethrin	80	100.0	±0.0		94	93.6	±1.4	
		Permethrin	80	75.0	±2.8	2.66E−15	98	19.4	±0.4	*1.00E+00*
		PBO + permethrin	80	97.5	±0.6		98	19.4	±0.4	
	Weija	Deltamethrin	80	16.3	±4.4	3.22E−05	96	8.3	±1.2	2.11E−05
		PBO + deltamethrin	80	43.8	±10.5		99	32.3	±1.8	
		Permethrin	80	12.5	±2.6	*6.79E*−*02*	99	5.1	±0.4	*3.28E*−*01*
		PBO + permethrin	80	7.5	±1.1		93	7.5	±1.4	
Central	Gomoa Obuasi	Deltamethrin	100	19.0	±1.7	1.16E−14	80	20.0	±1.8	1.79E−12
		PBO + deltamethrin	80	93.8	±1.6		86	90.7	±1.7	
		Permethrin	80	3.8	±1.1	1.88E−08	85	1.2	±0.5	4.56E−08
		PBO + permethrin	80	38.8	±3.9		94	33.0	±2.4	
	Twifo Praso	Deltamethrin	80	36.3	±1.4	3.54E−07	73	13.7	±1.5	8.78E−06
		PBO + deltamethrin	80	82.5	±4.1		71	52.1	±3.4	
		Permethrin	80	21.3	±1.9	3.00E−10	90	5.6	±0.5	2.81E−04
		PBO + permethrin	80	76.3	±1.9		81	25.9	±3.4	
Eastern	Akuse	Deltamethrin	80	37.5	±3.6	1.2E−09	93	8.6	±2.1	1.1E−16
		PBO + deltamethrin	80	97.5	±0.6		92	91.3	±1.7	
		Permethrin	80	51.3	±1.4	1.8E−06	90	31.1	±1.6	1.8E−06
		PBO + permethrin	80	98.8	±0.5		93	72.0	±2.1	
	Kade	Deltamethrin	80	65.0	±8.8	4.65E−04	87	13.5	±0.9	9.93E−09
		PBO + deltamethrin	80	100.0	±0.0		83	72.8	±2.3	
		Permethrin	80	17.5	±0.6	2.45E−13	86	1.2	±0.5	3.46E−03
		PBO + permethrin	80	85.0	±0.9		80	12.5	±1.3	
Volta	Nkwanta	Deltamethrin	80	82.4	±3.0	*7.90E*−*02*	87	47.1	±1.4	7.80E−05
		PBO + deltamethrin	80	100.0	±0.0		83	90.4	±2.6	
		Permethrin	80	27.5	±1.9	1.35E−08	82	17.1	±1.5	3.77E−04
		PBO + permethrin	80	77.5	±4.9		71	46.5	±3.6	
	Afife	Deltamethrin	80	76.3	±1.1	*7.07E*−*02*	83	43.4	±6.4	1.28E−06
		PBO + deltamethrin	80	93.8	±1.1		85	92.9	±0.6	
		Permethrin	80	10.8	±4.2	2.18E−07	83	31.3	±2.8	*3.94E*−*01*
		PBO + permethrin	80	45.9	±2.8		83	26.5	±1.6	
Western	Prestea	Deltamethrin	80	75.0	±0.9	1.24E−02	88	13.6	±0.8	1.47E−15
		PBO + deltamethrin	80	100.0	±0.0		88	97.7	±0.9	
		Permethrin	80	91.3	±0.5	*3.82E*−*01*	81	27.2	±4.9	7.04E−10
		PBO + Permethrin	80	100.0	±0.0		88	85.2	±0.9	
	Sefwi Wiawso	Deltamethrin	80	25.0	±2.4	1.16E−13	72	30.6	±3.8	6.14E−07
		PBO + deltamethrin	80	98.8	±0.5		80	75.0	±1.9	
		Permethrin	80	15.0	±2.0	2.66E−15	76	11.8	±2.3	5.69E−05
		PBO + permethrin	80	90.0	±2.0		83	37.3	±3.2	

*PBO* piperonyl butoxide, *CI* confidence interval, *nd* could not be determined, the numbers in italics showed the non-significant p values

**Table 2:** Mortality of *Anopheles gambiae s.l.* exposed to deltamethrin and permethrin alone as well as PBO + deltamethrin and PBO + permethrin in 2015 and 2016 in the northern sector

Regions	Sentinel site	Insecticide	2015				2016			
			Total tested	% Mortality	CI 95% mortality	*p* value	Total tested	% Mortality	CI 95% mortality	p value
Nothern	Sawla	Deltamethrin	77	18.2	±2.1	9.24E−11	96	7.3	±0.4	3.29E−16
		PBO + deltamethrin	79	84.8	±2.1		95	84.2	±2.4	
		Permethrin	79	15.2	±0.9	1.58E−03	94	5.3	±1.1	1.61E−02
		PBO + permethrin	80	36.3	±1.0		95	14.7	±0.9	
	Wulensi	Deltamethrin	80	27.5	±1.4	2.29E−10	83	0.0	±0.0	5.55E−13
		PBO + deltamethrin	78	100.0	±0.0		83	62.7	±2.5	
		Permethrin	82	42.7	±2.7	9.25E−05	89	3.4	±1.0	1.76E−07
		PBO + permethrin	80	83.8	±2.4		88	37.5	±2.0	
Ashanti	Konongo	Deltamethrin	78	12.8	±1.5	2.92E−10	89	1.1	±0.5	4.87E−15
		PBO + deltamethrin	79	73.4	±1.0		84	75.0	±2.2	
		Permethrin	nd	nd	nd	nd	83	13.3	±0.6	1.11E−09
		PBO + permethrin	nd	nd	nd		83	68.7	±1.0	
	Ejura	Deltamethrin	79	24.1	±1.9	1.62E−10	80	41.3	±0.9	5.18E−07
		PBO + deltamethrin	80	92.5	±2.1		78	98.7	±0.5	
		Permethrin	77	15.6	±2.4	1.61E−04	80	95.0	±1.3	*7.49E−01*
		PBO + permethrin	78	43.6	±3.8		78	100.0	±0.0	
Upper East	Zebilla	Deltamethrin	79	89.9	±1.5	3.68E−01	84	21.4	±1.3	1.35E−11
		PBO + deltamethrin	80	98.8	±0.5		91	84.6	±0.9	
		Permethrin	nd	nd	nd	nd	82	8.5	±0.9	1.44E−02
		PBO + permethrin	nd	nd	nd		85	20.0	±1.3	
	Fumbisi	Deltamethrin	95	20.0	±1.2	2.14E−12	81	49.4	±3.2	3.19E−06
		PBO + deltamethrin	88	94.3	±0.9		85	96.5	±0.9	
		Permethrin	84	42.9	±1.7	1.31E−06	90	17.8	±1.2	1.29E−04
		PBO + permethrin	84	94.0	±0.5		95	42.1	±1.8	
Upper West	Wecheau	Deltamethrin	84	20.2	±1.6	1.62E−09	84	34.5	±1.6	4.06E−08
		PBO + deltamethrin	87	75.9	±3.2		80	96.3	±0.5	
		Permethrin	82	10.4	±1.3	5.81E−07	87	10.3	±0.9	3.08E−13
		PBO + permethrin	83	49.4	±3.9		87	80.5	±1.4	
	Tumu	Deltamethrin	86	38.4	±0.5	5.32E−07	87	4.6	±1.3	1.05E−15
		PBO + deltamethrin	77	100.0	±0.0		84	85.7	±2.9	
		Permethrin	78	43.6	±4.0	5.46E−04	98	12.2	±2.1	1.16E−05
		PBO + permethrin	81	75.3	±0.6		91	42.9	±3.2	
Brong Ahafo	Kenyasi	Deltamethrin	84	17.9	±1.0	1.23E−05	91	70.3	±4.8	*6.49E−02*
		PBO + deltamethrin	84	52.4	±2.8		80	95.0	±1.3	
		Permethrin	84	7.1	±2.2	7.63E−03	87	63.2	±3.9	2.04E−02
		PBO + permethrin	83	20.5	±3.5		85	88.2	±0.7	
	Kwame Danso	Deltamethrin	78	12.8	±1.9	1.66E−13	84	36.9	±4.3	1.12E−08
		PBO + deltamethrin	83	88.0	±1.0		86	96.5	±1.0	
		Permethrin	89	21.3	±0.7	7.64E−07	82	13.4	±1.1	2.73E−06
		PBO + permethrin	88	63.6	±1.6		84	48.8	±5.3	

*PBO* piperonyl butoxide, *CI* confidence interval, *nd* could not be determined, the numbers in italics showed the non-significant *p* values

**Fig. 2:**
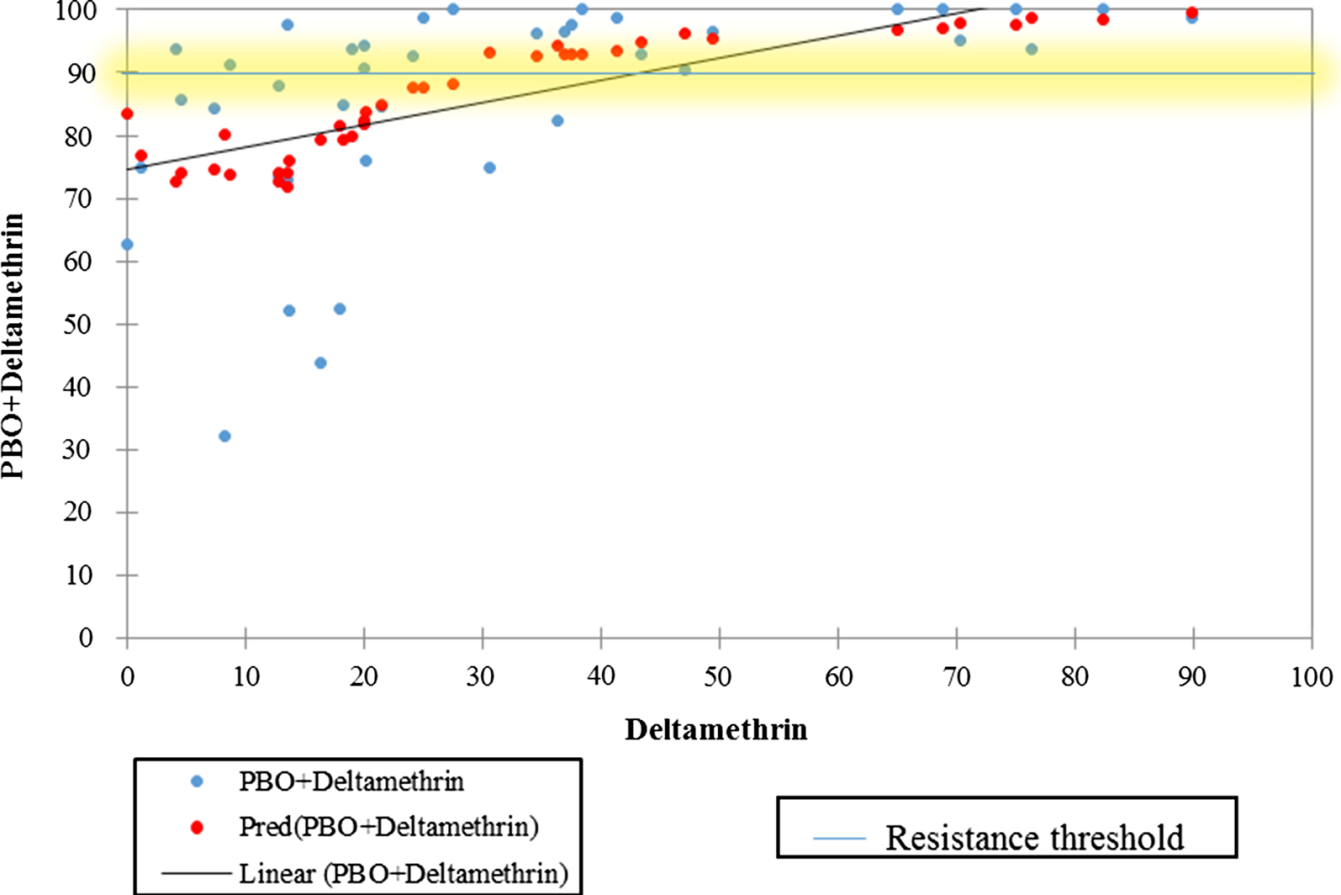
Trends and prediction of the percentage increment in susceptibility of *Anopheles gambiae s,l.* against PBO + deltamethrin in relation to deltamethrin alone during 2015 and 2016 for both Southern and Northern sectors. Significant increment in susceptibility was observed with the addition of PBO (p = 0.001)

In 2015, the average mortality of *An. gambiae s.l.* against deltamethrin alone in the southern sector was 50.2% for the 10 sites with the lowest mortality recorded at Weija in the coastal area in the Greater Accra region and the highest rate recorded at Nkwanta in the Volta region (82.4%). When the mosquitoes were pre-exposed to PBO before deltamethrin, there was a 45% increase in mortality with an average of 91% in all the sites (Fig. 2). The resistance status of mosquitoes tested was completely reversed to full susceptibility in more than five sites in the southern sector. Hundred percent (100%) mortality was observed at Ada, Kade, Nkwanta and Prestea whilst 99% was recorded at Sefwi Wiawso. The lowest mortality of mosquitoes to PBO + deltamethrin exposure was recorded in Weija (43.8%).

**Fig. 3:**
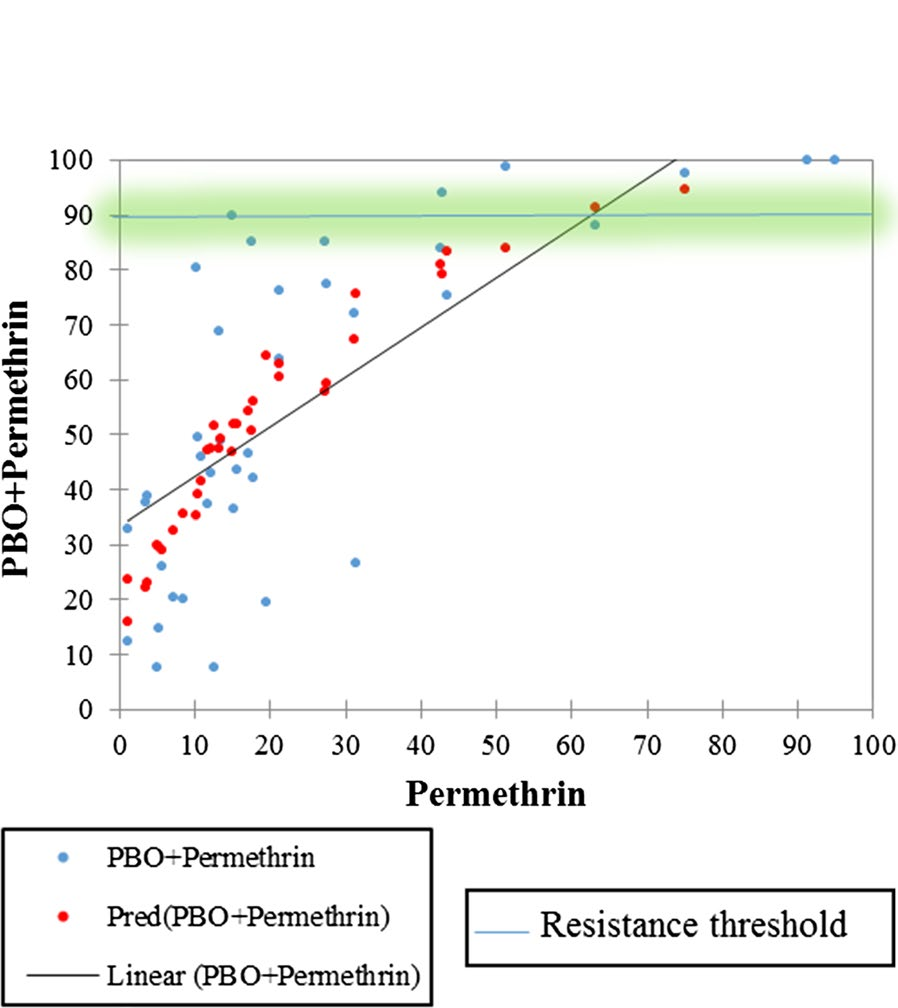
Trends and prediction of the percentage increment in susceptibility of *Anopheles gambiae s.l.* against PBO + permethrin in relation to permethrin alone during 2015 and 2016 for both Southern and Northern sectors. Significant increment in susceptibility was observed with the addition of PBO (p < 0.001)

The average mortality of *An. gambiae s.l.* when exposed to permethrin alone during the same year was 32.6% within the 10 sites in the southern sector. Half of the sites recorded less than 20% mortality indicating high permethrin resistance in this part of the country. Only Ada and Prestea showed a much higher mortality of 75 and 91.3% respectively. The lowest mortality of *An. gambiae s.l.* against permethrin was recorded at Gomoa Obuasi in the Central region (3.8%). Similarly to the deltamethrin in the same part of the country, pre-exposure of to PBO increased the mortality of the mosquitoes against permethrin to an average of 71% (Fig. 3). Moreover, Weija still recorded a low non-significant mortality of 7.5% after pre-exposure to PBO compared to the permethrin alone.

In 2016, there was an increase in deltamethrin resistance compared to the previous year. An average of 20.3% mortality was recorded across the 10 southern sites with a lowest rate of 4.1% at Ada and the highest at Nkwanta (47.1%). A significant enhancement of susceptibility to deltamethrin was observed during both years after pre-exposure to PBO. An average of 79% mortality was recorded in 2016. However, the lowest mortality was recorded at Weija (32.3%).

Permethrin resistance was also high across all the sites in 2016 with an average mortality of 15%. Mosquitoes in 80% of the sites in the southern sector recorded less than 20% mortality against permethrin alone, with a low mortality observed at Gomoa Obuasi and Kade (1.2%). The average mortality was enhanced two times for permethrin after pre-exposure of mosquitoes to PBO. The average mortality recorded for all the sites was 36.6% with the highest of 85.2% occurring at Prestea (Table 1).

In the northern sector, the mortality of *An. gambiae s.l.* against deltamethrin in 2015 ranged from 12% at Konongo in the Ashanti region to 89% at Zebilla in the Upper East region (Table 2). The average mortality of mosquitoes against deltamethrin was 28.2% and susceptibility was enhanced three times to 86% averagely after pre-exposure to PBO. The lowest rate was recorded at Kenyasi (52.4%) while the highest rate of 100% was recorded at Wulensi and Tumu in the Northern and Upper West regions respectively. Similar trends were observed with permethrin and PBO + permethrin with 24.9 and 58.3% as average mortalities, respectively.

In 2016, the average mortality of mosquitoes against deltamethrin was 26.7% for all the sites. No mortality was recorded at Wulensi in the Northern region and as low as 1.1% was recorded in Konongo in the Ashanti Region (Table 2). The highest mortality of 70.3% was recorded at Kenyasi. When the mosquitoes were first pre-exposed to PBO, the mortality against deltamethrin was increased more than three times with an average rate of 87.5% in all the sentinel sites. The trends observed were almost the same as in 2015. There was also a significant (*p* < *0.001*) increase in mortality of *An. gambiae s.l.* to permethrin during the same year after pre-exposure to PBO (average mortality 23.2% for permethrin alone vrs 54.3% for PBO + permethrin).

Furthermore, no significant difference was observed in mortality between both years for deltamethrin insecticide (*p* = *0.909* for deltamethrin alone and *p* = *0.061* for PBO + deltamethrin). Similarly, no difference was observed between permethrin alone and PBO + permethrin during the 2 years (*p* = *0.141* permethrin alone and *p* = *0.236* for PBO + permethrin).

In contrast, there is a significant difference between insecticides. Deltamethrin alone is significantly more effective than permethrin alone (*p* = *0.007*) and PBO + Deltamethrin was more effective than PBO + permethrin (*p* < *0.001*) (Figs. 2 and 3).

## Discussion

Pyrethroid resistance has in recent years become widespread among anopheline mosquitoes in Western, Eastern, Central and South Africa [5, 6, 8, 20–32]. In spite of that, most of the malaria vector control programme of countries is still relying on pyrethroid treated tools to control the vectors [1, 33–37]. The results of this study are in line with other studies carried out in some areas in Ghana [5, 8–10, 20]. This is a cause for concern because the use of ITNs and indoor residual spraying (IRS) form the main vector control tools in the country. Pyrethroids are the insecticide of choice for ITNs/LLINs. It is known that cross resistance between permethrin and DDT does occur, so although DDT is not used in the country for public health, permethrin resistance is particularly high and that may explain the trend that was observed at all the sites [38, 39]. These observations raise the possibility that mass use of insecticide-treated bed nets for malaria control could be rendered ineffective if the vectors are already resistant to pyrethroids [3, 40, 41]. However, the impact of the insecticide resistance of *An. gambiae s.l.* on the vector control tools such as LLINs need to be well described to delimit the real effects of pyrethroid resistance on the effectiveness of all the mass distribution of long-lasting insecticidal nets across countries [42, 43].

The study also found that when the mosquitoes were pre-exposed to PBO before exposure to deltamethrin or permethrin, there was a significant enhancement of susceptibility of *An. gambiae* s*.l.* from almost all the sentinel sites. Weija in the southern region and Kenyasi in the northern sector showed a non-significant difference of 24 h mortality both in year 2015 and 2016 after pre-exposure to PBO. The other sites encountering a non-significant effect of the PBO were due to the high susceptibility level of the mosquitoes in those sites. Among those sites are Nkwanta, Afife and Prestea in the southern regions and Zebilla in the northern region. PBO is one of many synergist which when added to insecticides can increase their lethality, or more generally their effectiveness against insect pests [15, 44–46]. PBO is known to enhance the effects of several insecticides, including the pyrethroid deltamethrin and this is achieved by inhibiting metabolic enzyme defense systems, such as P450 s within the insect [16, 17]. The results of these synergist assays carried out in 2015 and 2016 indicate that metabolic enzyme activities such as oxidases could be involved in the development of resistance to the pyrethroids and other insecticides tested. This means that vector control products that incorporate this synergist may be useful in killing *An. gambiae s.l.* even in the presence of pyrethroid resistance. However, the ability of PBO to synergize pyrethroid insecticides seems to diminish at higher levels of resistance as observed at Weija in the southern region. A similar trend was observed in an initial meta-analyses reported by Churcher et al. using bioassay data collected from different experimental hut trials of PBO-LLINs [47]. Such trends need to be more described in order to understand the implications on vector control strategies. The study also recommends that the involvement of other enzymes apart from P450s in the development of resistance across the sentinel sites needs to be investigated as to understand the full influence of biochemical mechanisms.

## Conclusion

Pyrethroid resistance was high in all the sites surveyed and therefore a rational use of insecticides especially pyrethroids should be encouraged in the country whilst promoting strategies that can help reduce the insecticide pressure. The enhancement of deltamethrin and permethrin susceptibility with PBO indicates that vector control products such as LLINs that have the synergist incorporated can be effective in killing mosquitoes even in the presence of pyrethroid resistance.
